# The role of tobacco smoking and illicit drug use in adolescent acute alcohol intoxication

**DOI:** 10.1186/s12887-021-02710-3

**Published:** 2021-05-17

**Authors:** Loes de Veld, Inge M. Wolberink, Joris J. van Hoof, Nico van der Lely

**Affiliations:** 1 Erasmus University, European School of Health Policy and Management, Burg. Oudlaan 50, 3062 Rotterdam, PA The Netherlands; 2 Department of Paediatrics, Reinier de Graaf Gasthuis, Reinier de Graafweg 5, 2635 Delft, AD The Netherlands; 3 Faculty of Behavioural, Management and Social Sciences, University of Twente, PO Box 217, 7500 Enschede, AE The Netherlands; 4 Faculty of Medicine and Health Sciences, University of Antwerp, Universiteitsplein 1, WILRIJK, 2610 Antwerpen, Belgium

**Keywords:** Adolescence, Alcohol intoxication, Combined illicit drug use, Cannabis, Tobacco use

## Abstract

**Background:**

This study aims to determine the prevalence of tobacco smoking and illicit drug use among Dutch adolescents admitted to hospital for acute alcohol intoxication treatment. Furthermore, socio-demographic predictors for smoking and illicit drug use in the sample population will be studied. The relationship between illicit drug use and specific characteristics of intoxication, such as blood alcohol concentration (BAC) and duration of reduced consciousness is also investigated.

**Methods:**

The national Dutch Paediatric Surveillance Unit was used to prospectively register cases of acute alcohol intoxication from 2007 through 2017. Cases were included if they met the following inclusion criteria: BAC > 0.0 g/L, aged between 10 to 18 years old and requiring hospital treatment due to reduced consciousness. Questionnaires were sent to paediatricians to obtain clinical information.

**Results:**

During the period 2007–2017, 5322 cases that met the inclusion criteria were reported. In this patient group, the prevalence of tobacco smoking was 22.2% (CI 21.0–23.5%), while the prevalence of illicit drug use was 11.8% (CI 10.9–12.7%). The predictors for smoking were the absence of alcohol-specific parental rule-setting, lower educational level, non-traditional family structure and positive drug screening. The predictors for illicit drug use were the absence of alcohol-specific parental rule-setting and smoking. Illicit drug use was also associated with a lower BAC at the time of admission.

**Conclusions:**

Assessing smoking and illicit drug use among adolescents admitted for acute alcohol intoxication is important in acute cases of intoxication, for outpatient follow-up and for the purposes of prevention. The relationship between simultaneous illicit drug use and a lower BAC is of relevance for paediatricians’ attempts to diagnose acute intoxication. With respect to outpatient follow-up and preventive measures, it is important to be aware that adolescents’ alcohol consumption, tobacco and illicit drug use are related and, ultimately, increase the odds of using other substances.

## Introduction

Harmful health behaviours, such as smoking tobacco, consuming alcohol and using illicit drugs typically commence during adolescence [1–3]. Several studies suggest that the initiation of sensation-seeking and risk-taking behaviour is triggered by tension between, on the one hand, the early development of subcortical regions that express exaggerated reactivity to motivational stimuli, and, on the other, the later maturation of the prefrontal regions which are associated with regulatory control and risk assessment [1, 4, 5]. This imbalance in the maturation of brain regions is enhanced by peer pressure, which is known to diminish cognitive control and, in turn, lead to adolescents being at increased risk of impulsive behaviour and experimenting with substance use [6, 7]. Truancy and runaway behaviour in adolescence have also been identified as predictive factors for binge drinking, alcohol dependence, illicit substance use and poor general life satisfaction in late adolescence and young adulthood [8].

The combined use of alcohol and illicit drugs has been found to be associated with various short-term deleterious health consequences. The use of illicit drugs alone has been associated with increased healthcare engagement, namely in the form of increased emergency department episodes and hospital admissions [9]. The co-ingestion of alcohol and cocaine can potentiate the cardio toxic effects associated with both cocaine and alcohol [10], which serves to increase the risk of immediate death as a result of the hepatic metabolism of cocaethylene [11]. The combined use of (meth) amphetamines and alcohol decreases alcohol-specific feelings of intoxication, such as feeling drunk and sedated, resulting in more severe alcohol intoxications [12]. The combination of alcohol and other sedatives, such as gamma-Hydroxybutyric acid (GHB), increases the risk of reduced consciousness, respiratory depression and admission to an intensive care unit [13, 14]. In adolescents who were not intoxicated, the simultaneous use of cannabis and alcohol was associated with the use of higher quantities of both substances than when either substance was used concurrently or alone [15–17]. Among adolescents, the combined use of alcohol and drugs has also been associated with violence and aggression [18], trauma [19], involvement in cyberbullying [20] and sexual risk behaviour [21].

Preventing combined alcohol and drug use among adolescents is of critical importance in the long-term, due to the fact that the onset of most cases of substance use disorders occurs during adolescence [22, 23]. Adolescents who have engaged early in regular smoking and drunkenness-orientated alcohol use, are particularly at risk of developing hazardous substance use later in life [24–26]. Indeed, a recent review indicates that alcohol and tobacco potentiate each other’s rewarding effects, and, hence, that concurrent usage may potentiate their respective negative effects [27]. In adulthood, alcohol and tobacco use are highly comorbid and have multiplicative health risks when used in conjunction with one another. The concurrent use of alcohol and tobacco, in comparison to both alcohol use and tobacco use alone, have been associated with supra-multiplicative health risks, such as cardiovascular problems, head and neck cancers, cirrhosis, pancreatitis and psychiatric comorbidity [28–30]. Another study indicated that from midlife onwards, age-related decline in the global cognitive score was faster in individuals who were smokers and heavy drinkers than in non-smoking moderate drinkers, which suggests that the combined effects of smoking and alcohol consumption are greater than their individual effects [31].

Despite the short- and long-term negative consequences of polysubstance use, strong associations between alcohol use, tobacco usage and illicit drug use have been established [32, 33]. In Europe, almost all students (87% or more) who used a licit or illicit substance also reported having consumed alcohol, while 93% of students who ever smoked cigarettes also consumed alcohol [34]. Similar associations have been found in the Netherlands, where the prevalence of cannabis use among adolescents who had tried alcohol was 21%, in comparison to 1% among adolescents who had never tried alcohol [35].

Although prior research has demonstrated the strong associations between alcohol usage and the use of other substances across the general adolescent population in the Netherlands, the simultaneous use of tobacco or illicit drugs by adolescents admitted to hospital for acute alcohol intoxication has hitherto not been explored. This study aims to identify both the socio-demographic predictors and deleterious effects of the combined use of tobacco and illicit drugs among Dutch adolescents admitted to hospital for acute alcohol intoxication. We hypothesised that, just like has been demonstrated in the general adolescent population, smoking and illicit drug use are strongly related and important determinants for each other.

## Materials and methods

### Study population and data collection

In 2007, the Dutch Paediatric Surveillance Unit (NSCK), which was initiated by the Dutch Paediatric Society, started collecting data on acute alcohol intoxication. The purpose of the surveillance system is to, firstly, gain population-level insights into the prevalence of rare and new diseases among youths (0–18 years), and secondly, to promote scientific research that addresses the background, nature and prognosis, as well as the treatment and prevention, of these diseases. Approximately 90% of Dutch paediatricians report to the system if they diagnose a disease included in the surveillance system. Data collection by the NSCK was approved by the medical ethical committee of the Faculty of Behavioural, Management and Social Sciences, University of Twente. All adolescents provided their informed consent and additional parental informed consent was obtained for participants younger than 16 years of age. Cases were reported to the system if they met the following two major inclusion criteria: blood alcohol concentration (BAC) > 0.0 g/L and under 18 years of age. With respect to the present study, only those admissions that pertained to reduced consciousness were included (admissions for different reasons, such as aggression, vomiting, suicide attempts and injuries were excluded from the analyses).

### Outcome measures

This study aimed to determine the prevalence of tobacco smoking and illicit drug use among the study population. Smoking was defined as a dichotomous variable, based on the current smoking status of the participant (either smoking or non-smoking). Due to its availability and societal acceptance, alcohol and tobacco smoking, are the psychoactive substances with the highest consumer rates worldwide [36]. Therefore, alcohol use and tobacco smoking are often classified as separate entities with the psychoactive substances. Illicit drug use was also defined as a dichotomous variable: negative drug screening and positive drug screening. Drug screening was based on self-reported declarations, heteroanamnesis and clinical signs that were suggestive of illicit drug use. According to protocol, admission for acute alcohol intoxication was an indication for a urine toxicology test and the results of those urine toxicology tests were used to confirm self-reported declarations and clinical signs. Illicit drug use was coded in accordance with the categories listed in the routinely used urine toxicology test: cannabinoids, cocaine metabolites, (meth) amphetamines (including 3,4-methylenediocymethamphetamine) and GHB. There was one residual category “other” that pertained to those drugs not in the above groups, such as mushrooms, nitrous oxide and opioids.

### Covariates

Subsequent to reporting to the surveillance system, paediatricians received instructions and a questionnaire in order to collect data on general patient characteristics (such as age at time of admission and sex), demographic characteristics (such as educational level, ethnicity and family structure), intoxication characteristics (such as BAC and duration of reduced consciousness) and substance use patterns prior to this instance of acute intoxication (tobacco smoking, alcohol consumption and illicit drug use). Completion of the questionnaire required conducting a standardised interview with the adolescents admitted for acute alcohol intoxication, and gathering details from their patient records, such as laboratory results.

Educational level was defined as a categorical variable comprising three categories, which corresponded to the Dutch secondary school system: low (pre-vocational education), middle (senior general secondary education) and high (pre-university education). Family structure was defined as a categorical variable made up of two categories: traditional family structure (both biological parents) and non-traditional family structure (all other family structures, such as, for example, divorced parents, single-parent households, or foster care). Alcohol-specific parental rule-setting was defined as a categorical variable consisting of the following categories: zero-tolerance rule-setting, partial permission to consume alcohol and the absence of alcohol-specific parental rule-setting (drinking allowed).

### Statistical data analysis

IBM SPSS Statistics (IBM Corp. Released 2017/ IBM SPSS Statistics for Windows, version 25.0, Armonk, NY: IBM Corp) was used for all the statistical analyses. Continuous variables were expressed as means and standard deviation. Categorical variables were expressed as frequencies with 95% confidence intervals (CIs).

First, the prevalence of tobacco smoking and illicit drug use were determined via the use of descriptive statistics. A binomial logistic regression was performed to ascertain the effects of age group, sex, educational level, ethnicity, family structure and alcohol-specific parental rule-setting on the likelihood of participants currently smoking or using illicit drugs. A Bonferroni correction was applied using multiple terms in the model.

## Results

During the period 2007–2017, 5322 cases that met the inclusion criteria were reported to the system. Smoking status was reported in 94.7% of the cases, while the drug screening results were reported in 90% of the cases. The mean age of the adolescents admitted for acute alcohol intoxication was 15.4 years (SD 1.2 years). The prevalence of tobacco smoking and illicit drug use is displayed in Table 1. Overall, 22.2% (CI 21.0–23.45%) of the adolescents admitted for acute alcohol intoxication smoked cigarettes. The prevalence of illicit drug use among adolescents admitted for acute alcohol intoxication was 11.8% (CI 10.9–12.7%), with cannabis being the most frequently consumed illicit drug. Table 2 shows the prevalence of smoking and illicit drug use in various demographic subgroups, as well as presenting the results of the logistic regression model.

**Table 1:** Prevalence of smoking and illicit drug use, NSCK 2007–2017

	Prevalence 2007–2017 (CI)	n
Smoking status		4789
Smoking	22.2% (CI 21.0–23.4%)	1063
Illicit drug use		5041
Illicit drug use	11.8% (CI 10.9–12.7%)	549
*Cannabis*	*6.8% (CI 6.1–7.5%)*	342
*Cocaine*	*0.3% (CI 0.1–0.5%)*	13
*(Meth)amphetamine*	*0.7% (CI 0.5–1.0%)*	38
*GHB*	*2.1% (CI 1.7–2.5%)*	104
*Other type of drugs*	*0.8% (CI 0.6–1.3%)*	42
*Multiple*	*1.1% (CI 0.8–1.4%)*	55

**Table 2:** Socio-demographic predictors for smoking and illicit drug use, NSCK 2007–2017

	Smoking		Illicit drug use	
	Prevalence	Adjusted OR	Prevalence	Adjusted OR
**Sociodemographic factors**				
Age category				
≤ 14 years (ref)	19.9% (CI 17.7–22.4%)	1.00	10.8% (9.2–12.8%)	1.00
15–16	22.1% (CI 20.6–23.7%)	1.26 (CI 0.86–1.86)	11.4% (10.3–12.6%)	1.29 (CI 0.82–2.01)
17–18	25.6% (CI 25.6–28.7%)	1.38 (CI 0.80–2.39)	14.3% (12.1–16.8%)	1.76 (CI 0.96–3.20)
Sex				
Males (ref)	22.5% (CI 20.8–24.2%)	1.00	13.6% (CI 12.3–15.0%)	1.00
Females	21.8% (CI 20.2–23.6%)	0.79 (CI 0.58–1.07)	10.0% (CI 8.8–11.2%)	0.74 (CI 0.52–1.05)
Educational level				
Low (ref)	28.6% (CI 26.5–30.7%)	1.00	12.8% (CI 11.3–14.5%)	1.00
Middle	15.0% (CI 12.9–17.3%)	0.50 (CI 0.35–0.71)***	9.7% (CI 8.1–11.7%)	1.11 (CI 0.75–1.66)
High	9.9% (CI 8.1–12.1%)	0.31 (CI 0.20–0.49)***	7.9% (CI 6.2–9.9%)	0.74 (CI 0.45–1.20)
Ethnicity				
Native Dutch (ref)	22.0% (CI 20.8–23.4%)	1.00	11.3% (CI 10.4–12.3%)	1.00
Other ethnic background	23.5% (CI 20.0–27.4%)	0.78 (CI 0.47–1.30)	14.3% (CI 11.5–17.6%)	1.43 (CI 0.86–2.39)
Family structure				
Traditional family structure (ref)	9.9% (CI 20.8–23.4%)	1.00	9.9% (CI 8.9–11.0%)	1.00
Non-traditional family structure	18.1% (CI 20.0–27.4%)	1.43 (CI 01.05–1.96)*	16.3% (CI 14.5–18.3%)	1.42 (CI 1.00–2.02)*
Alcohol-specific parental rule-setting				
Zero-tolerance rule-setting (ref)	16.7% (CI 14.5–19.1%)	1.00	11.5% (CI 9.7–13.6%)	1.00
Partial permission	18.5% (CI 15.3–22.2%)	1.08 (CI 0.73–1.57)	13.6% (CI 10.8–16.9%)	1.03 (CI 0.68–1.56)
Parental approval	27.1% (CI 23.4–31.2%)	1.38 (CI 0.91–2.08)	12.2% (CI 9.6–15.3%)	0.89 (CI 0.54–1.45)
**Substance use patterns**				
Current smoking status				
Non-smoking (ref)			7.1% (CI 6.3–8.0%)	1.00
Smoking			26.6% (CI 24.0–29.4%).	4.21 (CI 2.92–6.06)***
**Intoxication characteristics**				
Illicit drug use				
No illicit drug usage (ref)	18.4% (CI 17.2–19.6%)	1.00		
Illicit drug usage	51.5% (CI 47.2–55.9%)	4.26 (CI 3.00–6.2)***		
Blood alcohol concentration		0.74 (CI 0.55–0.98)*		0.59 (CI 0.43–0.82)**
Year of admission (2007–2017)		0.88 (CI 0.80–0.97)**		1.04 (CI 0.93–1.16)

*** *p* < .001,** 0.001 < *p* < .01, * 0.01 < *p* < 0.05

The logistic regression model for tobacco smoking was statistically significant, χ2(12) = 152.6, *p* < .001. The model correctly classified 83.4% of the cases. Of the predictors, five determinants were statistically significant: educational level, family structure, BAC, illicit drug use and year of diagnosis. Low educational level was associated with an increased likelihood of smoking, in comparison to adolescents with a middle or high educational level. Being raised in a non-traditional family structure increased the odds of smoking by a factor of 1.43 (CI 1.05–1.96, *p = .02*) in comparison to adolescents raised in a traditional family structure, with both biological parents. Positive drug screenings were associated with 4.26 (CI 2.97–6.13, *p < .001*) times higher likelihood of tobacco smoking than adolescents who had a negative drug screening. During the study period 2007–2017, the likelihood of smoking decreased each year by a factor of 1.14 (CI 1.04–1.24, *p =* .007). Increasing BAC at admission was associated with an decreased likelihood of smoking (OR 0.74, CI 0.55–0.98, *p =* .04).

The logistic regression for illicit drug use was also statistically significant, χ2(12) = 102.1, *p* < .001. The model for illicit drug use correctly classified 88.0% of the cases. Three determinants were statistically significant: family structure, smoking and BAC. The odds of a positive drug screening were 1.42 (CI 1.00–2.02, *p = .*05) times higher among adolescents raised in a non-traditional family structure, compared to adolescents raised in a traditional family structure. Among adolescents admitted for acute alcohol intoxication, smoking was associated with 4.21 (CI 2.96–6.06, *p <* .001) higher odds of illicit drug use than non-smoking. Increasing BAC was associated with a decreased likelihood of illicit drug use (OR 0.59, CI 0.43–0.82, *p = .002*).

The results of the descriptive statistics for the different types of illicit drug use are presented in Table 3. The results indicate that among Dutch adolescents admitted for acute alcohol intoxication, the prevalence of illicit drug use was slightly higher among male adolescents (*X*^*2*^ *(1, n = 5012) = 15.8, p < .001)*. However, this difference appears to be related to the increased prevalence of cannabis consumption among male adolescents compared to female adolescents (*X*^*2*^ *(1, n = 5012) = 29.2, p < .001)*. Furthermore, a positive urine drug screening for cannabis or (meth) amphetamines was associated with a lower BAC at admissions (ANOVA (6, *n* = 4566) = 11.5, *p < .001*, post hoc analyses *p < .001).* The combined use of alcohol and GHB seems to be associated with a lower BAC at admission too, however, the difference is not significant (*p* = 0.36), most likely due to a too small sample size for sub analyses. A positive urine drug screening for cocaine-metabolites seems to be associated with a higher BAC at admission, but the sample size is too small to test this. In the analyses of the association between the subgroups of illicit drug use and BAC, it is important that age at admission might act as a confounder. However, the sample sizes are too small to correct for age using a multivariable linear regression analysis.

**Table 3:** Descriptive statistics for illicit drug use, NSCK 2011–2017

	None (ref)	Cannabis *n* = 342	Cocaine *n* = 13	(Meth)amphetamine *n* = 38	GHB *n* = 104	Polysubstance > 2 *n* = 55
Sex						
Prevalence in males	86.4%	8.7%	0.3%	0.8%	1.7%	1.3%
Prevalence in females	90.0%	4.8%	0.2%	0.7%	2.4%	0.9%
Mean age at admission	15.4	15.4	16.0	16.0	15.3	15.8
BAC	1.95	1.77	2.02	1.48	1.81	1.74
Duration of reduced consciousness	3.0	3.3	2.6	1.8	3.2	3.1

## Discussion

This study has shown that approximately one fifth (22.2%) of the adolescents admitted for acute alcohol intoxication were active smokers, while approximately one eighth (11.8%) of the adolescents had a positive drug screening. According to a World Health Organization collaborative cross-national survey examining the health behaviour of school-aged children, the prevalence of smoking (that is, whether they had smoked in the month prior to the survey) among 15-year-olds declined from 27.4% in 2009 to 14.0% in 2017 [34]. The prevalence rate of 6.8% of positive urine screenings for cannabis appears to be in line with the prevalence of cannabis use among 15-year-olds in the general Dutch adolescent population (ranging from 8.2% in 2013 to 12.6% in 2005 [34]). The results thus indicate that smoking and illicit drug use are common among adolescents admitted for acute alcohol intoxication.

Although the prevalence of illicit drug use did not appear to be higher in adolescents admitted for acute alcohol intoxication than it is for the general adolescent population, the results of this study demonstrate why it is of vital importance to assess adolescents’ smoking status and illicit drug use. Firstly, the assessment of illicit drug use is important in instances of acute intoxication, insofar as this study has demonstrated that the simultaneous consumption of alcohol and various illicit drugs (i.e. cannabis, GHB, (meth)amphetamines) is associated with a lower BAC at admission. This result suggests that in comparison to alcohol alone, simultaneous use of these substances results in admission for reduced consciousness at lower BACs. Furthermore, assessing smoking and illicit drugs is important for the follow-up of adolescents admitted for acute alcohol intoxication. Specifically, our study shows that among adolescents admitted for acute alcohol intoxication, smoking increased the odds of having a positive drug screening and, moreover, that having a positive drug screening increased the odds of smoking. Smoking was also associated with higher quantities of regular alcohol use during the weekend. These results show that during adolescence, the use of various substances, such as alcohol, tobacco and illicit drugs are intertwined.

Literature review provides insights in multiple concepts that address polysubstance use in adolescents. Recent neuroscience models of adolescent brain development attribute the morbidity of this period to structural and functional imbalances between more fully developed limbic regions that subserve reward and emotion as opposed to the frontal cortex that enables cognitive control [5, 37–39]. The “imbalance model” describes a peak in sensation seeking and impulsive behaviour during adolescence, which produces more risk taking behaviour than in children or adults [5, 37–39]. In contrast to the “imbalance model”, “Life-span wisdom models” consider the role that experience plays in healthy adolescent development [5, 40]. The “life-span wisdom models” describe a peak in sensation seeking during adolescence motivates greater exploration in ambiguous environments, but risk taking declines monotonically from childhood to adulthood when risks are known, per greater reliance on gist and increasing executive function. Socioemotional influences can promote risk taking, but social experience and positive social influences can promote healthy risk avoidance [5, 41]. Other models have been used to address specific types of adolescent risk-taking behaviour: the involvement with psychoactive substances. The “gateway model” focusses on the sequence of drug initiation and considers drug itself as the cause of drug use development. The model states that there is a progressive and hierarchical sequence of stages of drug use that begins with tobacco or alcohol, two classes of drugs that are legal, and proceeds to cannabis, and from cannabis to other illicit drugs, such as cocaine or (meth) amphetamines [40, 42, 43]. Whereas the “gateway model” does not specify mechanistic connections between “stages”, and does not extend to the risks for addictions, the concept of “common liability to addictions” incorporates sequencing of drug use initiation as well as extends to related addictions and their severity [40]. Liability denotes a latent (unobservable) quantitative trait that, when measured, “would give us a graded scale of the degree of affectedness or of normality” [44]. The quantity of models reflect researchers eagerness to find options for treatment and prevention of polysubstance abuse in adolescents and therefore, a lot of research has been conducted to risk factors of alcohol use, smoking and illicit drug use.

Our study identified educational level, family structure and alcohol-specific rule-setting as predictors for smoking in adolescents with acute alcohol intoxication. Educational attainment, as indicated by both years of education and level of education, has had a consistent inverse relationship with drug use and drug use problems [45, 46]. Alcohol and drug-related problems have been identified as important predictors of negative school-related outcomes, such as low grade point average and high levels of hours missed from school [47]. Family factors, such as family structure and alcohol-specific rule-setting have been identified as important modifiable factors in adolescent substance abuse [48–50]. Health risk factors for adolescent substance abuse can be classified in various categories: genetic, environmental and personal determinants [51]. Identification of risk-factors of polysubstance abuse among adolescents is essential, as identification of risk-factors form the basis in the development of prevention strategies against negative outcomes of polysubstance abuse.

A recent study among young adults with experience in the simultaneous use of alcohol and cannabis showed that cross-fading motives (i.e. to enhance the effects of either alcohol or cannabis, or to get drunk and high at the same time) are common. In this study, the existence of greater cross-fading motives was associated with greater alcohol use and increased perceived intoxication [52]. In a study examining the perceived acute effects of alcohol use, cannabis use, and simultaneous alcohol and cannabis use, most effects (i.e. clumsiness, confusion, dizzyness and difficulty concentrating) were rated strongest when that person was engaging in simultaneous use, compared to typical alcohol and cannabis use alone [53]. The lower BAC among adolescents with acute alcohol intoxication and positive urine screening for cannabis in comparison to alcohol alone can perhaps also be explained by a pharmacokinetic study, which showed that the simultaneous use of alcohol and cannabis produces significantly higher blood concentrations of the main psychoactive constituent of cannabis, Δ9-tetrahydrocannabinol (THC) [54].

Our study demonstrated that a positive drug screening for (meth) amphetamine was associated with a relatively low BAC at admittance. In order to interpret these study results, it is important to realise that most urine toxicology screenings used in the Netherlands fail to distinguish between 3,4-mythylenedioxymethamphetamine (MDMA), methamphetamines and amphetamines. A pharmacokinetic study showed that co-ingestion of MDMA and alcohol resulted in a 13% increase in the MDMA plasma concentration and a 9 to 15% decrease in the ethanol plasma concentration [55]. The combined use of MDMA and alcohol has also been associated with a dissociation between subjective and objective sedation [55, 56]. The effects associated with the co-ingestion of ethanol and MDMA may depend on several factors, including the interval between dosing, ethanol dosage and MDMA dosage [57]. The pharmacokinetics of MDMA, combined with the dissociation between subjective and objective sedation, might contribute to the relatively lower BAC of adolescents with acute alcohol intoxication who simultaneously use (meth)amphetamine. In a small-scale study examining the acute and residual interactive effects of repeated administrations of oral methamphetamine and alcohol, there was no difference found in the breath alcohol levels between the placebo + amphetamine group and the methamphetamine + alcohol group. Co-administration of methamphetamine and alcohol produced greater feelings of euphoria and good drug effects than single doses of either drug alone. The drug combination decreased alcohol-specific feelings of intoxication, such as feeling drunk and sedated [58]. The study also noted that the reduction of alcohol-specific feelings increased the risk of higher BAC and alcohol intoxication [58].

Previous studies have shown that the co-ingestion of ethanol increases the adverse effects experienced by patients intoxicated from GHB, in turn, leading to greater depression of consciousness, need for treatment and admission to intensive care units [13, 14]. In comparison to co-ingestion of GHB alone, alcohol co-use was associated with increased risk of showing agitation and vomiting [13, 15]. The combination of cocaine and alcohol can lead to the production of cocaethylene, which is more lethal than cocaine itself [10, 11]. Alcohol has been shown to increase the plasma concentration of cocaine [59]. Consumption of both cocaine and alcohol has also been found to increase the heart rate and systolic blood pressure [59]. Cerebral hypoperfusion was shown to be more common among individuals using both alcohol and cocaine, compared to those who used cocaine and alcohol in isolation [60]. Assessing the illicit drug use of adolescents admitted for acute alcohol intoxication is thus necessary for preventing substance use later in life, as research has shown a strong continuity between substance use in adolescence and young adulthood [61].

## Conclusions

Assessing smoking and illicit drug use among adolescents admitted for acute alcohol intoxication is important, insofar as illicit drug use increases the odds of smoking and smoking increases the odds of illicit drug use. This study has demonstrated that smoking is associated with higher quantities of regular alcohol use during weekends. Therefore, in the treatment of adolescents admitted for acute alcohol intoxication, smoking and illicit drug use should serve as a warning for health care professionals, while appropriate attention should also be paid to smoking and illicit drug use in outpatient follow-up and when designing preventive measures. The predictors identified by this research for smoking among adolescents with acute alcohol intoxication were lower educational levels, non-traditional family structures and the absence of alcohol-specific parental rule-setting. The latter was also a predictor for simultaneous usage of alcohol and illicit drugs.

## Data Availability

The datasets used and/or analysed during the current study are available from the corresponding author on reasonable request.

## Abbreviations

BAC: Blood alcohol content
NSCK: Dutch Paediatric Surveillance Unit/Nederlands Signalerings Centrum Kindergeneeskunde
MDMA: 3,4-Methylenedioxymethamphetamine
GHB: γ-Hydroxybutyric acid
